# Confronting the Challenges of Anatomy Education in a Competency-Based Medical Curriculum During Normal and Unprecedented Times (COVID-19 Pandemic): Pedagogical Framework Development and Implementation

**DOI:** 10.2196/21701

**Published:** 2020-10-07

**Authors:** Nerissa Naidoo, Aya Akhras, Yajnavalka Banerjee

**Affiliations:** 1 College of Medicine and Health Sciences Mohammed Bin Rashid University of Medicine and Health Sciences Dubai United Arab Emirates; 2 Centre of Medical Education University of Dundee Dundee United Kingdom

**Keywords:** undergraduate medical education, anatomy education, Gagne’s 9 events of instruction, Peyton’s 4-step approach, Mento’s 12-step change management model, Bourdieu’s Theory of Practice, social media application, interactome, COVID-19, framework

## Abstract

**Background:**

Anatomy is considered to be one of the keystones of undergraduate medical education. However, recently, there has been drastic reduction, both in gross anatomy teaching hours and its context. Additionally, a decrease in the number of trained anatomists and an increase in the costs associated with procuring human cadavers have been noted, causing a diminution of cadaveric dissections in anatomy education.

**Objective:**

To address these challenges, there is an ardent need for a pedagogical framework such that anatomy education can be disseminated through active learning principles, within a fixed time frame, using a small team of anatomists and a small number of cadaveric specimens (for live on-site sessions) as well as collaborative learning principles. The latter is particularly important when anatomy education is delivered through distance learning, as is the case currently during the COVID-19 pandemic.

**Methods:**

Here, we have blueprinted a pedagogical framework blending the instructional design models of Gagne’s 9 events of instruction with Peyton’s 4-step approach. The framework’s applicability was validated through the delivery of anatomical concepts, using an exemplar from the structure-function course Head and Neck during the normal and COVID-19–mandated lockdown periods, employing the archetype of Frey syndrome. Preliminary evaluation of the framework was pursued using student feedback and end-of-course feedback responses. The efficiency of the framework in knowledge transfer was also appraised.

**Results:**

The blueprinted instructional plan designed to implement the pedagogical framework was successfully executed in the dissemination of anatomy education, employing a limited number of cadaveric specimens (during normal times) and a social media application (SMA)–integrated “interactome” strategy (during the COVID-19 lockdown). Students’ response to the framework was positive. However, reluctance was expressed by a majority of the faculty in adopting the framework for anatomy education. To address this aspect, a strategy has been designed using Mento’s 12-step change management model. The long-term benefits for any medical school to adopt the blended pedagogical framework have also been explicated by applying Bourdieu’s Theory of Practice. Additionally, through the design of an SMA interactome model, the framework’s applicability to the delivery of anatomy education and content during the ongoing COVID-19 pandemic was realized.

**Conclusions:**

In conclusion, the study effectively tackles some of the contemporary key challenges associated with the delivery of anatomy content in medical education during normal and unprecedented times.

## Introduction

Anatomy education is an essential stipulation for medical students, general practitioners, surgeons, and for all those involved in invasive diagnostic and therapeutic procedures [1]. In the recent years, numerous factors are disadvantageously impacting anatomy education in medical specialties. These factors include, but are not limited, to a drastic reduction in anatomy teaching hours and its context and the number of trained anatomists, as well as an increase in the costs of human cadaveric dissections and the related ethical uncertainties surrounding the use of human cadavers.

The COVID-19 pandemic has added to these challenges, as most medical schools have suddenly shifted from face-to-face teaching to distance learning, requiring the design of innovative strategies that will allow for the delivery of remote anatomy education [2].

One way of effectively addressing these challenges is to design a “student-centered teaching framework” (easily implementable for both face-to-face and distance-learning modalities), where the essential “nuts and bolts” of anatomy can be delivered effectively: (1) within a limited and fixed time frame; (2) using a small team of trained anatomists; (3) using a small number of cadaveric specimens; and (4) by integrating principles of active learning, collaborative learning, feedback, and student autonomy.

Moreover, designing a pedagogical framework alone will not address the challenges of anatomy education. The designed teaching approach needs to be implemented in the delivery of anatomy education and then evaluated. Furthermore, a change management strategy needs to be adopted such that the pedagogical framework is able to initiate a change in pedagogical philosophy in the context of anatomy education.

Here, we outline a pedagogical framework to tackle the aforementioned challenges of anatomy education in a competency-based medical curriculum (CBMC). A pedagogical framework was designed, blending Gagne’s [3] and Peyton’s [4-6] instructional design models. We have also demonstrated how this pedagogical framework can be effectively employed in the delivery of anatomical concepts using an exemplar from the structure-function course Head and Neck offered to first-year medical students in the preclinical phase of the undergraduate medical curriculum at the Mohammed Bin Rashid University of Medicine and Health Sciences (MBRU). Further, we have outlined a social media application (SMA)– integrated strategy (an SMA interactome), whereby the designed pedagogical framework could be employed in anatomy education during the COVID-19 pandemic. The efficiency of this solution in terms of knowledge transfer was evaluated by comparing the performance of the cohorts who were exposed to the pedagogical framework: (1) through face-to-face teaching and (2) through distance learning during the COVID-19 lockdown. A preliminary evaluation with regards to student perceptions toward the pedagogical framework was also conducted based on end-of-course feedback. Although the students responded positively to the pedagogical framework for both face-to-face and distance learning modalities, there was reluctance among instructors in adopting the framework for anatomy education across all anatomy courses. To address this, we have blueprinted a change-management approach employing Mento’s change-management model [7], which will allow anatomy educators to implement the designed pedagogical framework in any CBMC. This paper primarily focuses on the description of the frameworks, and initial observations and reflections following their execution.

## Methods

### Study Landscape

**Figure 1:**
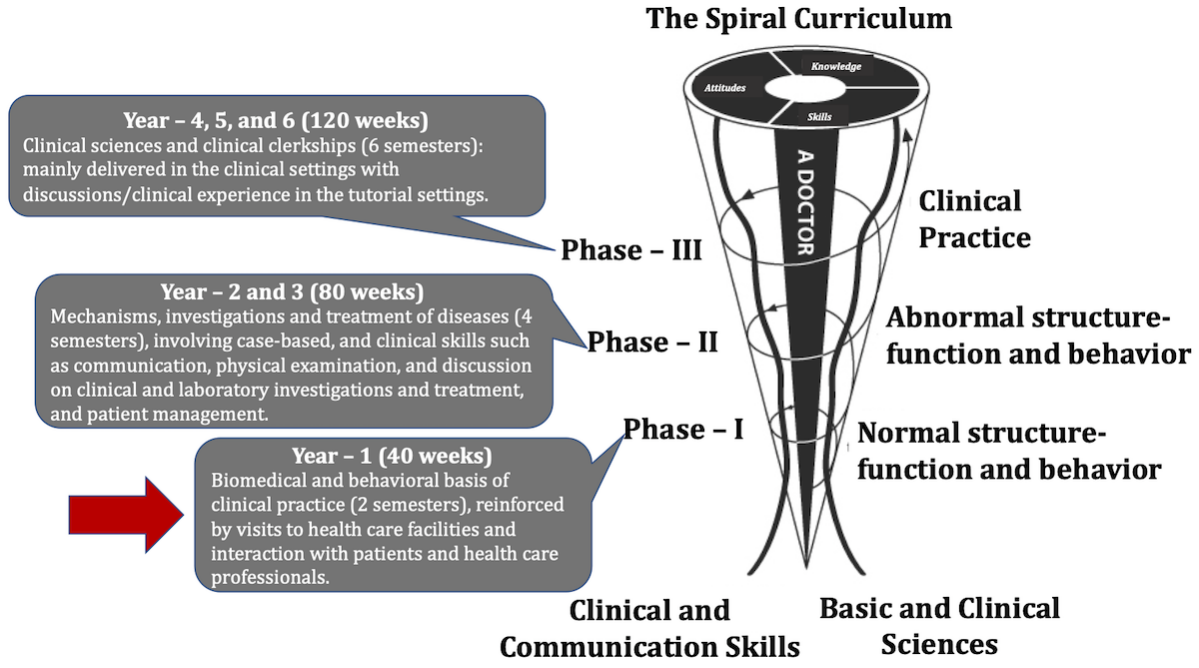
The undergraduate medical curriculum at Mohammed Bin Rashid University of Medicine and Health Sciences (MBRU). The curriculum is divided into three phases and spans over 6 years. Note: Each phase of the undergraduate medical curriculum includes integrated courses and builds on the preceding one, such that the curriculum is a “spiral,” and the students repeat the study of a subject, each time at a higher level of difficulty and in greater depth. The phase in which the teaching framework was implemented is indicated with a red arrow.

The CBMC at MBRU comprises three phases (Figure 1). Each phase of the curriculum includes integrated courses and builds on the preceding one, such that the curriculum is a “spiral” [8,9], and the students repeat concepts relating to a subject, where with each successive encounter, concepts build on the previous one. The medical school caters to a student population from more than 19 different countries and from 20 different high school curricula. Approximately 75% of the students are female [10]. The designed pedagogical framework was implemented in the delivery of gross regional anatomy in the form of structure-function courses occurring primarily in Phase 1 of the curriculum (Figure 1). Four structure-function courses with specific timelines are delivered in semester 2 of Phase 1 over a 15-week period: (1) Limbs and Spine: weeks 1-4; (2) Thorax: weeks 5-7; (3) Abdomen, Pelvis and Perineum: weeks 8-11; and (4) Head and Neck: weeks 12-15.

The structure-function courses are designed to provide students with in-depth understanding of the normal human anatomy and resulting physiological roles, with a focus on essential radiology and basic clinical correlation. The structure-function course Head and Neck where the pedagogical framework was implemented provides students with functional knowledge of the structures of head and neck regions that will enable further understanding of organ system courses in Phase 2 (Figure 1). The course also introduces the concept of “living anatomy of the head and neck” as visualized on conventional medical imaging and on the living human body. At the end of the course, students should be able to describe the major features of the skull, as well as the main structures present in the neck, face, and temporal and infratemporal regions. They should also be able to identify the main anatomical features of the face, nose, oral cavity and tongue, pharynx, soft palate, and larynx, and explain the basis of cranial nerve testing. They should also be able to explain the anatomical basis of upper airway obstruction, cervical swellings, facial nerve palsy, Frey syndrome, epistaxis, and dysphagia. In addition, through the course, students should develop an attitude of collaborative learning and autonomy.

### Design of the Pedagogical Framework

In order to design the pedagogical framework, we employed the instructional design models of Gagne [3] and Peyton [4,11]. Gagne’s 9-step instructional model is based on a behaviorist approach to learning, whereas Peyton’s 4-step approach avails a task-centered approach. Our pedagogical framework availed a blended approach similar to that of Tambi et al [10], which allowed us to disseminate both cognitive and noncognitive skills. These models were selected based on a pilot study conducted at MBRU, where the learning approaches of MBRU students were mapped using the Approaches and Study Skills Inventory for Students (ASSIST) learning approach investigation tool [12]. The pilot study indicated that most MBRU students avail deep/strategic learning approaches, suggesting that they favored constructivist learning approaches or strategies [12]. Therefore, we adopted Gagne’s and Peyton’s instructional design models to create our pedagogical framework, since these models support deep/strategic learning approaches [6,13-15].

## Results

### Implementation and Design of the Pedagogical Framework

**Figure 2:**
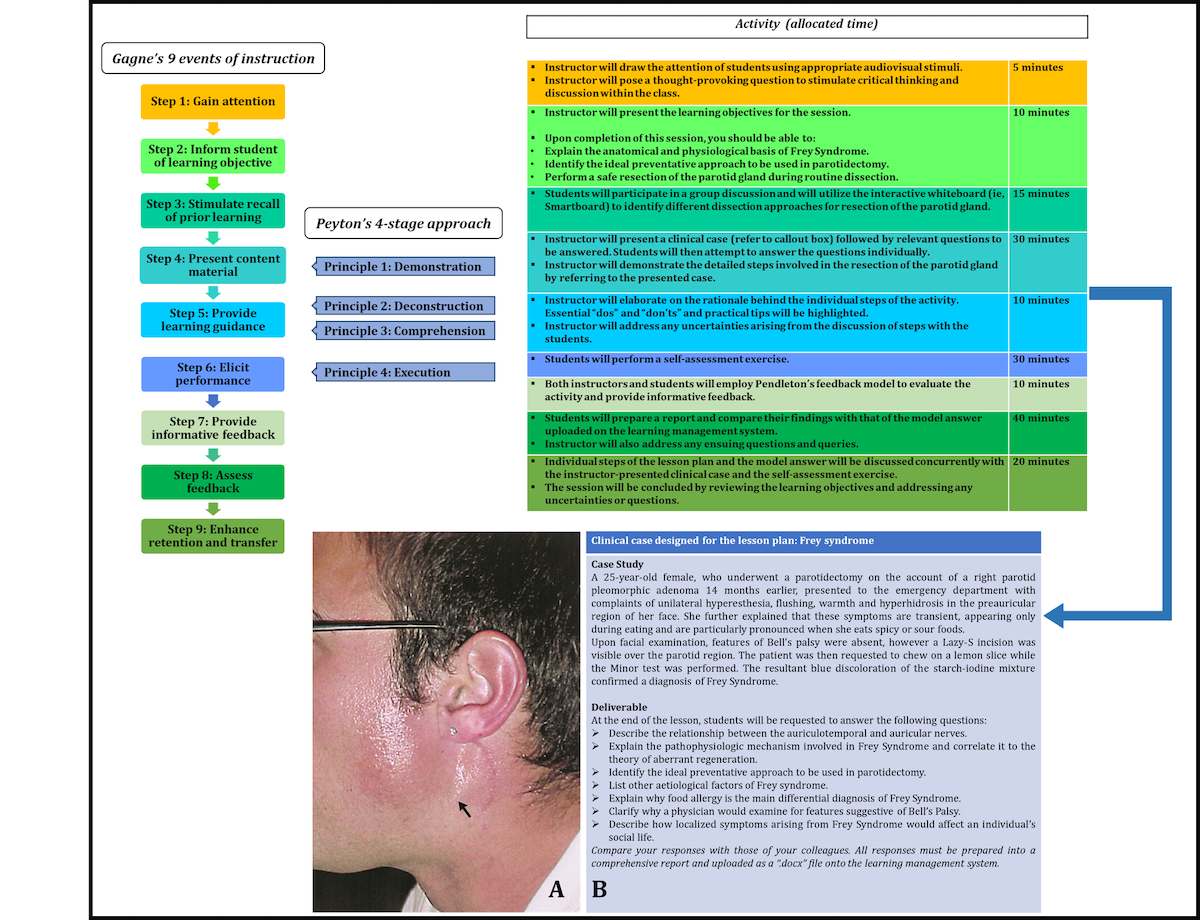
Design of the pedagogical framework. The instructional design strategies of Gagne and Peyton were blended to design the framework. The blended steps are also indicated. On the far right, the dissemination of the teaching framework with the sequential steps is shown with the allocated time for each step. The clinical case associated with Frey syndrome and the associated deliverables are shown. The medical image of Frey syndrome was adopted from Prattico and Perfetti [16] with permission.

The individual steps of the instructional plan associated with the pedagogical framework are described below (Figure 2).

#### Learning Environment

**Figure 3:**
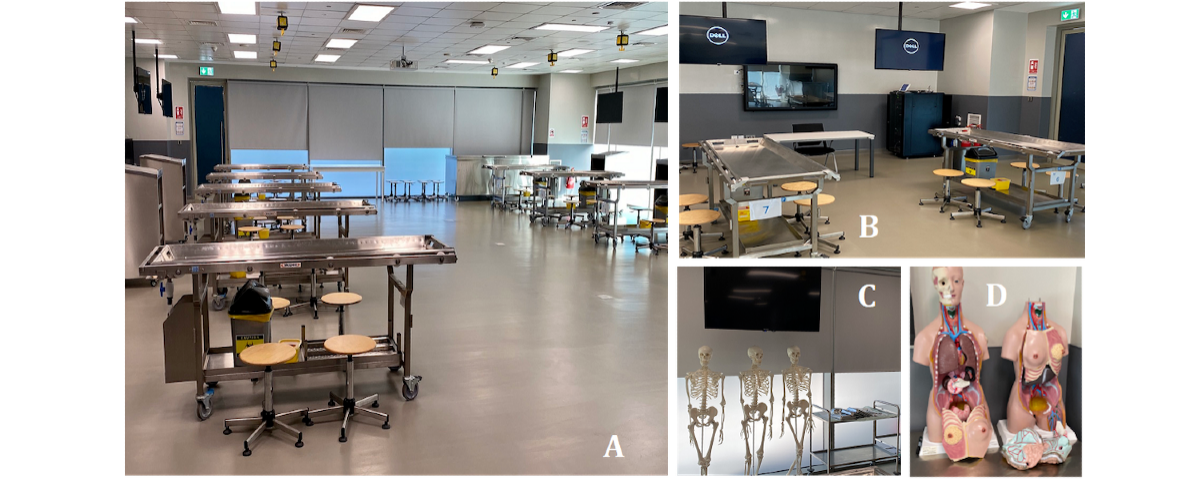
Anatomy dissection facilities at the Mohammed Bin Rashid University of Medicine and Health Sciences. (A) Dissection area showing the dissection stations (each station comprises an adult cadaver placed in the supine position on a removal tray situated on the dissection table); (B) medical imaging and case-based discussion area; (C) and (D) prosection areas.

For the implementation of the instructional plan, the anatomy dissection hall was chosen (Figure 3). The dissection hall is a well-lit rectangular room situated on the ground floor, with floor-to-ceiling windows spanning the length of two perpendicular walls. It consists of two dedicated teaching areas: the dissection area and the medical imaging and case-based discussion area (Figure 3).

#### Prerequisites

In preparation for the session, students were requested to review the following concepts: (1) boundaries and contents of the parotid region and (2) the structural and functional aspects of the course and distribution of the facial nerve. In addition, for the instructional plan, we decided to use the exemplar of the clinical case associated with Frey syndrome. In line, by reviewing the above concepts, we believed students would be better prepared to tackle the questions accompanying the case.

Learning material in the form of PowerPoint slides, medical images, and reading material pertaining to the above concepts were uploaded to the learning management system [17] 1 week prior to the session. These prerequisites enabled students to be adequately prepared for the session to successfully execute the tasks outlined in each step of the instructional plan. The activities and time frame pertaining to each step of the instructional plan are depicted in Figure 2.

#### Dissemination of Individual Steps of the Instructional Plan

The steps were tailored employing a “blended” methodology in which Gagne’s instructional model was integrated with Peyton’s 4-step approach (Figure 2).

##### Step 1: Draw Students’ Attention

The instructor applied the “pattern interrupt phenomenon” [18] to draw students’ attention. The resounding ring of a doorbell was used as the sudden auditory stimulus. This was followed by the Socratic method of delivery [19], whereby the instructor posed the question: “How would you describe Frey’s Syndrome to your younger brother?” A video available on The Doctors TV titled *Frey’s Syndrome* [20] was also shown to the students. This technique concurrently addressed visual, auditory, and kinesthetic learning styles [21,22].

##### Step 2: Inform Students About Learning Objectives

Students were then provided with set learning objectives, which they were expected to achieve at the conclusion of the instructional plan (Figure 2).

##### Step 3: Stimulate Recall of Prior Learning

Students participated in a group discussion to determine and evaluate the safest dissection approach when resecting the parotid gland. This enabled students to revise concepts related to gross, variational, functional, and living anatomy and helped them appreciate how these contributed to the accurate interpretation of imaging anatomy, safe clinical practice, and successful surgical outcomes. This step touched on the theories of multiple concepts [23], social learning [10], and team-based learning [24], as it incorporated peer-assisted education into the instructional plan.

##### Step 4 (Blended): Present Content Material

Students were then presented with a clinical case (Figure 3). The detailed steps involving the resection of the parotid gland and identification of the intact facial nerve were summarized by means of a flowchart and presented as a PowerPoint presentation (Multimedia Appendix 1). Step 1 of Peyton’s model was integrated here, which involved a demonstration of the steps of the dissection procedure.

##### Step 5 (Blended): Provide Learning Guidance

Principles of steps 2 and 3 of Peyton’s 4-step model was integrated here. Interactive learning was emphasized in this step. The instructor explained the individual steps for the activity (ie, dissection procedure) and provided clarity on the rationale behind it. The instructor then analyzed each step thoroughly, highlighting the essential “dos” and “don’ts” and provided a few practical tips (Peyton’s Principle #2). Students were encouraged to ask questions to clarify any doubts. This was followed by a conceptual phase during which philological and kinesthetic learning styles were encouraged as students were entreated to elucidate each step of the dissection procedure, while the instructor followed the guidelines (Peyton’s Principle #3). Such a practice enabled students to articulate the dissection procedure gradually, concomitantly allowing the instructor to assess their understanding.

##### Step 6 (Blended): Elicit Performance

This step corresponds to Peyton’s Principle #4. In this step, students were provided with the opportunity to reinforce their learning through performance; therefore, a larger amount of time was allocated to this step. The class was divided into 12 groups (approximately 5 students/group). Each group was assigned a cadaver and a dissection station (5 cadavers in total). In their designated groups, students attempted to perform the dissection procedure of the parotid gland as described by de Ru et al [25]. Students executed the dissection steps sequentially, followed by a group discussion on the results to ensure accuracy. This facilitated peer-assisted learning as it incorporated elements of interaction and collaboration [26]. Additionally, this step allowed the students to practice skills associated with the dissection of the parotid gland. Such dedicated practice of procedural dissection skills has been shown to increase students’ confidence in anatomy education [27].

In this step, the student groups were also asked to address the questions listed under deliverables in the clinical case of Frey syndrome (Figure 2 [16]). Each group presented answers to one of the listed questions. Since the clinical case and deliverable were uploaded a week prior to the dissection session, students had an opportunity to prepare their responses. This fostered self-directed learning and student autonomy [28]. Additionally, some of the student groups presented their responses using research articles related to the questions. These presentations followed a guide plan similar to the 6D-Approach, a pedagogical framework previously designed by us [29].

##### Step 7: Provide Informative Feedback

Informative feedback was provided employing Pendleton’s feedback model [30]. In their own group, students were able to provide feedback to their peers (ie, peer feedback [31]). This activity aimed at refining the student’s own understanding of where things stand; a so-called “reality check” that concurrently provides a clear trajectory in terms of improving behaviors, attitudes, and skills. While students conducted their own peer-to-peer feedback within their designated groups, the instructor visited each group and provided individual assistance and instantaneous feedback such that it didn’t lead to any false assessment on the part of the student with regard to their own skills and abilities. Students were also advised to clarify and discuss any uncertainties and/or questions as they rise. To conclude this step, students provided feedback about the activity by addressing the following:

- What do you think went well?
- What do you think could be done differently?
- What could be further improved?
- How can this be achieved?

##### Step 8: Assess Performance

In this step, students prepared a reflective report on their dissection experience and how that experience helped them to better understand the anatomical changes associated with Frey syndrome. The students prepared their report using Gibbs’ reflective cycle framework [32]. The report was uploaded by students to the learning management system [17] and contributed 5% to the total assessment component of the Head and Neck course. This step encouraged students to think critically about the content disseminated, as well as improve their writing skills.

##### Step 9: Enhance Retention and Transfer

Following submission of the report, students were required to assess a clinical scenario similar to Frey syndrome (namely, facial nerve paralysis) using the sequential steps of the dissection procedure, which they were exposed to earlier [25], to grasp the learning activity, which concluded by reviewing the learning objectives and resolving any uncertainties.

### The SMA Interactome Strategy to Implement the Pedagogical Framework During the COVID-19 Pandemic

**Figure 4:**
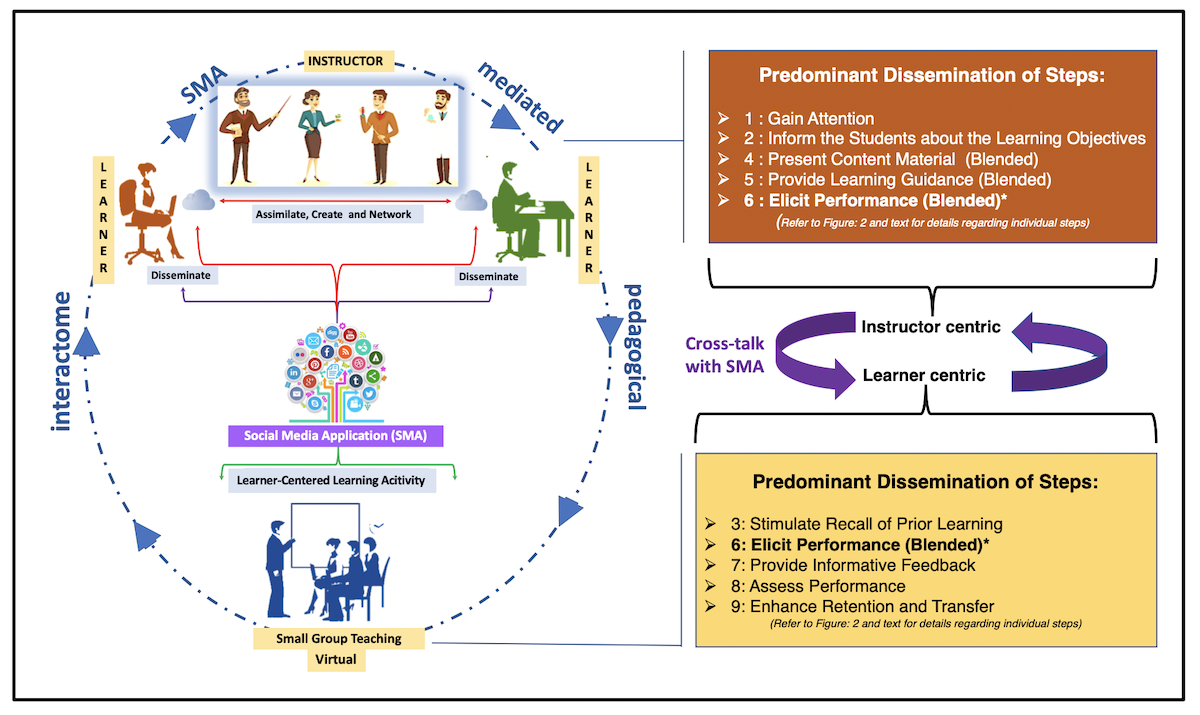
The SMA (social media application) interactome. The dissemination of the teaching framework using SMA during the unprecedented times of COVID-19 is shown. The interactome consists of two aspects, one of which is instructor centric whereas the other is learner centric. Individual steps of the teaching framework attesting to the two aspects in shown. Step 6 is common to both the aspects (indicated by *). The crosstalk between the two aspects is facilitated by SMAs (YouTube and WhatsApp at the Mohammed Bin Rashid University of Medicine and Health Sciences, and also through discussion sessions on the learning management system at the university).

Currently, with the COVID-19 pandemic sweeping across the globe, many medical schools have switched to the distance learning modality. So, we asked ourselves “Can our teaching framework adapt to this new pedagogical shift?” We have applied this framework again to the structure-function course Head and Neck, this time delivered through distance learning. To apply this framework, we designed an SMA-based “interactome” (Figure 4), such that different steps of the blended framework can be implemented using different SMAs. In fact, a pilot study at MBRU showed us that our students prefer the integration of SMAs such as YouTube and WhatsApp into their learning process [10]. Didactic sessions were delivered in the form of screencast using Microsoft Teams. For specific sessions, a flipped teaching approach was adopted, where students were provided with prerecorded lectures, which were uploaded to the learning management system at least a week prior to the session. In-session activities for sessions that adopted flipped teaching comprised of treatise focusing on the discussion of relevant clinical case(s) in small groups (consisting of 15-20 students in each discussion group), using the Microsoft Teams platform where the instructor (NN) was able to participate as well as moderate the discussion across several groups. Students were also encouraged to participate in discussions with their peers in designated WhatsApp group, which were created and moderated by the instructor. Such discussions primarily focused on tackling questions which could not be addressed in-depth during the SMA-integrated distance learning teaching sessions because of time constraints. Additionally, students were often directed to relevant podcasts and videos on YouTube, especially to demonstrate dissection procedures. For dissemination of our pedagogical framework during the COVID-19–mandated lockdown using the SMA interactome, we substituted the parotid gland dissection demonstration (which could not be conducted due to laboratory closures), with podcast videos available from different universities on YouTube [33]. The discussion associated with the clinical scenario of Frey syndrome was organized using Microsoft Teams and WhatsApp. Furthermore, for formative assessments, the instructor (NN) employed resources that were available from the University of Michigan [34].

### Efficiency of the Pedagogical Framework in Knowledge Transfer

The efficiency of the pedagogical framework in knowledge transfer was investigated by comparing the performance of students in the summative assessment of the Head and Neck course across three cohorts (Table 1): (1) a cohort where the course was delivered using traditional didactic pedagogy (n=58; mean score [out of 100] 64.9, SD 11.2); (2) a cohort where the course was delivered using the pedagogical framework but with the incorporation of dissection sessions (n=58; mean score 70.0, SD 11.6); and (3) a cohort where the course was delivered using the pedagogical framework but involved the use of the SMA interactome strategy (n=56; mean score 77.7, SD 11.1). As evident from Table 1 as well as the calculated mean scores, the implementation of the pedagogical framework in the delivery of anatomy education led to better performance, with the cohort that used the pedagogical framework along with SMAs having the highest scores (a mean score that was 19.7% higher than the control cohort). Furthermore, Kuder and Richardson formula 20 (ρ_KR20_) reliability values calculated for the multiple-choice question component (accounting for 75% of a typical summative assessment) for all the summative assessments of all three cohorts was higher than 0.75. This indicates that the summative assessments had high reliability, which further confirms our observation that implementation of the pedagogical framework in the delivery of anatomy education leads to better performance, and hence, augmented knowledge transfer. However, more dedicated studies are warranted to better understand the aspect of knowledge transfer. These future studies will focus on assessing learners’ perceptions of the pedagogical framework using validated tool and learning behaviors and styles of learners while being exposed to the framework.

**Table 1:** Performance of students in the summative assessment of the Head and Neck course across three cohorts: (A) cohort where the course was delivered on-site using traditional didactic pedagogy; (B) cohort where the course was delivered on-site using the blended pedagogical framework with incorporation of dissection sessions; and (C) cohort where the course was delivered using the blended pedagogical framework, with integration of the social media application interactome during the COVID-19–mandated lockdown period. Note: the performance of the students was better when the pedagogical framework was implemented in the delivery of anatomy education.

Cohort and range of % score		Students, n (%)
**A (n=58; average % score=65)**		
	31-41	2 (3)
	41-51	3 (5)
	51-61	12 (21)
	61-71	22 (38)
	71-81	16 (28)
	81-91	3 (5)
**B (n=58; average % score=70)**		
	42-53	6 (10)
	53-64	8 (14)
	64-75	22 (38)
	75-86	19 (33)
	86-97	3 (5)
**C (n=56; average % score=78)**		
	55.7-65.7	10 (18)
	65.7-75.7	14 (25)
	75.7-85.7	14 (25)
	85.7-95.7	18 (32)

### Preliminary Evaluation of Students’ Perceptions Toward the Pedagogical Framework

In the present work, our focus was on the design and implementation of the pedagogical framework. An elaborate evaluation of the perceptions of students toward the pedagogical framework is still pending and will be addressed in our future work. The evaluation presented here is only preliminary.

The pedagogical framework was evaluated informally following Pendleton’s approach [30] (Step 7 of the teaching plan). A measure with regard to the instructional plan’s ability to facilitate knowledge retention was obtained by reviewing the students’ reports at the conclusion of the course. We also reviewed the student feedback obtained at the end of the Head and Neck course.

The pedagogical framework was received positively by the students, who exhibited enthusiasm in both organizing and in participating in the event. Key points of note are as follows:

- Students from different academic backgrounds effectively functioned as a group.
- The reading habits of students improved significantly following their participation in the activity due to the increase in depth and content of the questions posed by the students during discussion. This observation is in line with the findings of Miner et al [35].
- Student autonomy was augmented, as many of them prepared concept/mind maps to correlate their understanding of the delivered concepts to their clinical significance.

Specific limitations that students believed need to be addressed are as follows:

- The time allocated for discussion (Step 6 in the instructional plan) was insufficient. The way to overcome this insufficiency is to integrate SMA into the delivery of the specific steps of the instructional plan, especially the ones that entail collaborative learning, similar to one of our previous studies [10].
- Students had difficulties accessing specific journals with regard to Step 6 of the instructional plan (since the institution didn’t have a subscription to these resources). One of the ways to side-step this limitation is to encourage students to refer to articles in open access journals of repute.

Formal student feedback for the Head and Neck course was obtained by using an institution-approved questionnaire for the cohorts where the pedagogical framework was implemented. The feedback for the course indicated that students expressed satisfaction with the instructional plan employed in the course;  79% (44/56) of students in both cohorts where the pedagogical framework was implemented strongly agreed with the highest grading score “extremely satisfied.” The majority of students (81/114, 71%) in both of the cohorts where the pedagogical framework was implemented indicated in open-ended comments that the instructional plan that was integrated into the Head and Neck course should be implemented across all structure-function courses in anatomy education, and if possible, especially in practical sessions involving dissection or discussion of clinical scenarios. Further, while evaluating the reports of the students, the instructor found that most students, while reflecting on their experience with regards to the instructional plan, identified that the pedagogical framework augmented their knowledge of anatomy pertaining to the session learning objectives, as well as helped them understand the clinical relevancy of the concepts.

## Discussion

In this study, we have blueprinted a pedagogical framework blending Gagne’s 9 events of instruction and Peyton’s 4-step teaching approach, and employed the framework in the dissemination of anatomy education both during normal and COVID-19–mandated periods. The framework was positively received by the students, who recommended its integration across all structure-function courses in Phase 1. Based on this feedback, the director of Phase 1 (YB) and the instructor who implemented the pedagogical framework (NN) approached other instructors in other structure-function courses to encourage the adoption of this framework. However, initial discussions indicated that instructors were reluctant to adopt the framework as it entailed elaborate modifications to their teaching approaches, which involved conformist strategies employed in anatomy education. However, this observation is not unique to our institution, and similar barriers have been encountered in medical education [36].

Accordingly, we decided to design a change management approach to integrate the pedagogical framework across all structure-function courses. This design involved the use of Mento’s change management model. We selected this model since it was previously used successfully to initiate change in pedagogical philosophy to implement active learning strategies in the medical curriculum, specifically in biochemistry and molecular biology courses [7]. The approach in which Mento’s model will be used in implementing the pedagogical framework in anatomy education is shown in Table 2. Details regarding the individual steps of Mento’s model have been discussed elsewhere; readers are requested to refer to Banerjee et al [7] for further information. We firmly believe that the versatility of both the pedagogical framework, and the proposed change management framework to implement it, will allow anatomy instructors to integrate the framework into any CBMC milieu.

**Figure 5:**
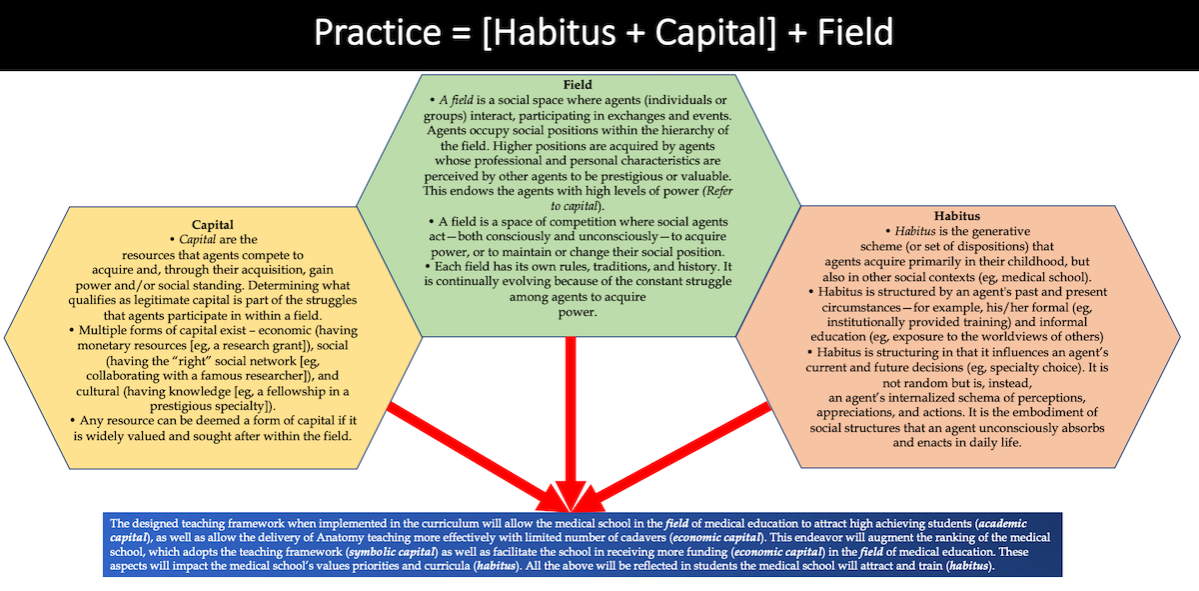
Bourdieu’s Theory of Practice. The figure elaborates on three intimately related concepts: field, capital, and habitus. The text box in blue elaborates how Bourdieu’s Theory of Practice when applied to the current context demonstrates the benefit of the teaching framework being adopted by a medical school. The concept of the figure was derived from Brosnan [37].

In addition, the benefit of the pedagogical framework being adopted by a medical school can be elaborated using Bourdieu’s Theory of Practice [37]. Bourdieu has developed three intimately related concepts: *field*, *capital*, *habitus* (refer to Figure 5 for details of the individual concepts). Applying Bourdieu’s Theory of Practice, the designed pedagogical framework, when integrated into a CBMC, will allow medical schools to attract high-achieving students (academic capital), as well as allow a more effective delivery of anatomy teaching with a limited number of cadavers (only 5 cadavers were used in the delivery of the teaching plan, whereas the ideal cadaver-to-student ratio at some of the top medical schools such as University of California, Los Angeles, and University of Washington is 5:1 and 4:1, respectively, therefore requiring 12 and 15 cadavers, respectively, for a similar student population) (economic capital). This endeavour will augment the ranking of the medical school, which has adopted the teaching framework (symbolic capital), as well as facilitate the school in applying and receiving more funding or emoluments (economic capital) in the field of medical education and health professions education research. These aspects cumulatively will impact the medical school’s values, primacies, and curricula (habitus). Furthermore, all the above will be reflected in the students the medical school will attract and train (habitus).

The fact that our pedagogical framework requires only a limited number of cadaveric specimens is pivotal, especially for medical schools in the Middle East where religion may play an imperative role in the number of cadavers available for dissection (Naidoo et al, unpublished data). Although Elamrani and colleagues [38] reason that, from a theological viewpoint, Islam does not prohibit dissection nor body donation, they posit that “the problem is actually cultural, societal and legislative and not religious.” Whatever the reason may be, the availability of cadavers for dissection in Middle Eastern medical schools is limited, and most schools import cadavers from the United States (usually donated bodies), from India (usually unclaimed bodies), or from the Philippines (source of bodies unclear) [39]. This is not only expensive, but also unwieldy (as apart from the price of the cadaver, there is a myriad of paperwork that needs to be tackled while cadavers are imported) [40]. In addition, importing cadavers also raise concerns about an international “trade” of dead bodies, with an often-debateable ethical foundation [41].

Apart from the above, body donation programs in many countries are also affected by local and political history [42,43]. For example, Kramer and Hutchinson [43] indicate that in South Africa, Black Africans are more disinclined than other ethnic groups to donate their bodies for medical education and research, which is not only related to their “cultural beliefs” but also to the country’s tumultuous “political history,” where the bodies of Black individuals were exploited for the education of White students. Analogous reasons may also be behind the qualms of African Americans toward body donation in the United States [42]. Therefore, our pedagogical framework will not only be beneficial for medical schools in the Middle East, but also for schools who want to integrate anatomy dissection into their curriculum but have limited access to cadavers.

Conventionally, anatomy is often perceived as an uninteresting, labor-intensive discipline, taught using surface-learning strategies and rote memorization [44]. Accordingly, students are often unable to translate how the anatomical concepts can inform their clinical practice, creating a so-called “integration gap” [45]. Our pedagogical framework integrates a real clinical scenario (the clinical scenario was developed around a real clinical case of Frey syndrome [16]) and implements student-centric active learning strategies. This will not only address the integration gap but also promote students to take an active role in learning and utilizing their own creativity, curiosity, and intelligence.

**Table 2:** Guidelines outlining the activities and timeline corresponding to each step of Mento’s change management model for the integration of a blended Gagne-Peyton instructional model in all structure-function courses.

Step	Mento’s model of change	Activity to facilitate/implement the change	Timeline
1	The idea and its context	Preliminary results from the HNSF^a^ course in Phase 1, semester 2, showed that the blended instructional model of pedagogy facilitates better learning in UME^b^. The idea is to integrate the blended instructional model throughout all structure-function courses in semester 2 of Phase 1.	N/A^c^
2	Define the change initiative	Present to concerned stakeholders the following: What are the attributes of the blended teaching approach of Gagne and Peyton? Benefits of the blended instructional model of Gagne and Peyton Planning of the teaching approach Successful case studies of the blended instructional model (eg, results of this study)	4 weeks prior to course initiation
3	Evaluate the climate for change	Assess the necessary resources, prior knowledge of stakeholders, and technological proficiency required to successfully implement the blended instructional model in the structure-function courses through SWOT^d^ analysis.	4 weeks prior to course initiation
4	Develop a change plan	Work with the technology-enhanced learning (TEL) and Smart Learning Hub (SLH) teams at MBRU^e^ to develop a faculty development plan to train stakeholders on the strategies to implement the blended instructional model of Gagne and Peyton in structure-function courses.	3 weeks prior to course initiation
5	Find and cultivate a sponsor	Schedule meetings with MBRU academic leadership (dean/associate deans/departmental chairs, phase directors) to inform them about the benefits of the blended instructional model and the resources required.	3 weeks prior to course initiation
6	Prepare your target audience	Organize faculty development workshops in collaboration with the TEL and SLH teams to inform stakeholders about *how* to implement the blended teaching approach in structure-function courses. Circulate nano-lectures on active learning to stakeholders over WhatsApp.	2 weeks prior to course initiation
7	Create a cultural fit	Create linkage between students’ learning approaches and the blended teaching approach to explain to concerned stakeholders *why* there is a necessity to create a culture of innovative pedagogy in UME.	2 weeks prior to course initiation
8	Develop and choose a lead team	Create an informal lead team consisting of the course coordinator and instructors of the HNSF course and digital advisors from the TEL and SLH teams, such that they can guide and encourage stakeholders to implement the blended teaching approach in the structure-function courses (at least 9 blended teaching sessions over 5 weeks).	1-5 weeks into the course
9	Create small wins for motivation	Identify the stakeholders who successfully integrated the blended teaching approach into their courses and request them to present their experiences in this effort to the MBRU academic leadership and other concerned stakeholders.	4-5 weeks into the course
10	Constantly and strategically communicate the change	During the whole transformation process: Create a “learning community” such that stakeholders can learn from each other about strategies to successfully implement the blended teaching approach in pedagogy. Try to address hurdles that are faced by stakeholders in their endeavours by communicating the change process to sponsors	1-5 weeks into the course
11	Measure the progress of the change effort	Refer to the updated pedagogical techniques of the concerned courses to appraise the number of teaching sessions where blended teaching was implemented. Evaluate the attitude of stakeholders toward blended teaching following the transformation initiative using an ADKAR^f^ framework. Assess the performance of the students in the structure-function courses to identify if blended teaching was beneficial over the traditional method. Obtain student feedback to assess students’ perceptions toward blended teaching.	6 weeks into the course following midterm assessments
12	Integrate lessons learned	Using a reflective framework conduct an After Action Review to: Map the transformation process Identify hurdles that need to be tackled such that blended teaching can be successfully integrated in other courses	6 weeks into the course following midterm assessments
Other notes		Preparatory time for implementing the transformation: 4 weeks Time required for implementing/assessing the transformation: 5 weeks Total study duration (preparation + implementation + assessment): 9 weeks	

^a^HNSF: Head and Neck structure-function course.

^b^UME: undergraduate medical education.

^c^N/A: not applicable.

^d^SWOT: strengths, weaknesses, opportunities, and threats.

^e^MBRU: Mohammed Bin Rashid University of Medicine and Health Sciences.

^f^ADKAR: awareness, desire, knowledge, ability, reinforcement.

Reflecting on our pedagogical framework against Harden’s integration ladder [46], we find that it attests to the correlation step of the ladder. Harden [46] postulates that curricular integration can be viewed as a ladder, with discipline-based teaching (isolation) at the bottom of the ladder and full integration (transdisciplinary teaching) at the top. Harden’s integration ladder has 11 steps from subject-based to integrated teaching and learning. In the first 4 steps (isolation, awareness, harmonization, and nesting) of the ladder, the emphasis is on the subjects or disciplines. As one climbs the ladder, the following 6 steps—temporal coordination, sharing, correlation, complementary, multidisciplinary, and interdisciplinary—underscore integration across multiple disciplines. In the final step (transdisciplinary), the students take responsibility for the integration and are given the tools to do so [46]. With regard to the correlation step in the ladder, an integrated teaching session is presented in addition to subject-based teaching. Our pedagogical framework attests to integrated teaching during dissection sessions; additionally, during in-class sessions, the instructor(s) can pursue subject-based teaching. Generally, in the early phases of a CBMC, integration is difficult to achieve [47]. The framework addresses this gap.

In recent times, anatomy teaching has undergone a paradigm change from “instructor-centered” to “student-centered” approaches [48-50]. Our teaching framework attests to this “paradigm change” as it mitigates two key challenges: (a) a dearth of trained anatomists for teaching anatomy (the dissemination of the framework requires only one trained anatomist [NN]); and (b) delivery of a large corpus of anatomy content within a limited time frame. These two benefits further advocate to Bourdieu’s economic capital (as anatomy concepts can be disseminated with a limited number of trained anatomists), simultaneously attracting academic high achievers (academic capital) to the medical school that adopts this framework.

During the mandated COVID-19 lockdown, we were able to implement the pedagogical framework through SMA integration. This further attests to the versatility of our teaching framework, which can be tailored according to the demands of a given situation. Of course, the detailed analysis with regard to students’ perception of this distance learning adoption of our pedagogical framework is still pending and will form the basis of our future studies.

### Limitations

Although our pedagogical framework has several inherent benefits as discussed above, it also has several limitations. Our pedagogical framework integrates only real dissection. However, studies have indicated that integrating real dissection and radiology using 3D image postprocessing tools provides a more enriching learning experience, as such a pedagogical strategy imparts familiarity with imaging and image postprocessing techniques and also improves anatomical understanding, radiological diagnostic skills, and 3D appreciation [51]. Will the presented teaching framework allow the blending of real dissection with virtual dissection within a limited duration of time? This aspect needs to be addressed. Unfortunately, MBRU is a new medical school, where the anatomy teaching team does not have a trained radiologist, which prevented us from addressing this question.

The dissemination of this pedagogical framework requires extensive instructor preparation, which may not allow instructors to adopt it, especially instructors who teach anatomy using conventional strategies. Our proposed change management framework may aid in mitigating this limitation.

The pedagogical framework integrates the precepts of peer-assisted learning (PAL) in several steps. However, this may be disadvantageous for some students, many of whom may feel they would learn better when they relate to the instructor. Additionally, students learning in a group can encounter problems, especially if they find themselves working with members in a group with whom they are not keen on collaborating. Furthermore, students working in a group may veer away from the point of an exercise and discuss irrelevant topics of interest. These aspects may be effectively addressed by involving peer tutors in the dissemination of the teaching framework.

Our framework was implemented in only one structure-function course, that too in the delivery of anatomy teaching. However, implementation of this framework across all structure-function courses may lead to cognitive overload [52], as our teaching framework necessitates students to adopt and practice self-directed learning.

A typical cohort at MBRU has 50 to 70 students. Dissemination of our pedagogical framework was successful with limited student numbers. However, many medical schools have 150 to 200 students in a cohort, and there is a possibility that this pedagogical framework may not work as effectively in such large cohorts. This may be because organizing group-based activities required for the implementation of the pedagogical framework with a larger cohort may be challenging.

Implementation of the framework requires instructor(s) to be conversant with the theoretical underpinnings of the instructional design models that were employed in blueprinting the framework. This may not be the case for all medical schools, especially the ones who use adjunct or part-time faculty members for the delivery of anatomy content. One way to address this gap will be to organize Continuing Professional Development modules for anatomy instructors, where the advantages of integrating the framework in anatomy teaching and the theoretical foundations of the framework can be elucidated.

In this study, we have provided the initial evaluation of our pedagogical framework. However, the detailed evaluation of this framework is still pending. This also raises the question, “What evaluation model will be best-suited to appraise the framework?” Our framework predominantly employs PAL at multiple steps, which functions on the theoretical foundation of social and cognitive congruence [53]. Based on this, we believe the teaching framework can be best evaluated by Stake’s Congruence-Contingency Model [54]. However, this needs to be explored further through dedicated studies. In addition, we can employ Kirkpatrick’s framework [55] to evaluate the pedagogical framework. However, this also warrants further long-term investigations.

### Conclusion

In conclusion, in this study we have delineated a pedagogical framework to teach anatomy during normal and unprecedented times, blueprinted using a blended approach exercising the instructional design strategies of Gagne and Peyton. The designed strategy integrates active learning principles and initiates a shift from the “sage on the stage” to “guide on the side” mode of delivery. Additionally, we have demonstrated the use of this framework in the successful delivery of anatomy concepts in a structure-function course in a CBMC both during normal and COVID-19 lockdown periods. Although our framework was well received by students, anatomy instructors at our medical school were reluctant to adopt the framework (a challenge that others may also face). To counter this, we propose a strategy designed using the change management model of Mento. We have also elaborated on the benefits to a medical school that adopts the pedagogical framework, which have been explicated through the use of Bourdieu’s Theory of Practice. We firmly believe that the delineated pedagogical framework will allow instructors to efficiently and effectively deliver concepts in anatomy education using cadaveric dissection or through the effective use of clinical scenarios, in a limited span of time, which will not only benefit students but will also be advantageous for the medical school.

## Appendix

Multimedia Appendix 1 PowerPoint presentation used in the delivery of the instructional plan.

## Abbreviations

ASSIST: Approaches and Study Skills Inventory for Students
CBMC: competency-based medical curriculum
MBRU: Mohammed Bin Rashid University of Medicine and Health Sciences
PAL: peer-assisted learning
SMA: social media application
