# Supplementary material for: Confronting the Challenges of Anatomy Education in a Competency-Based Medical Curriculum During Normal and Unprecedented Times (COVID-19 Pandemic): Pedagogical Framework Development and Implementation
Source: JMIR Med Educ. 2020 Oct 7;6(2):e21701. doi: 10.2196/21701 (PMC7546732; doi:10.2196/21701)
Supplement: Multimedia Appendix 1 [file mededu_v6i2e21701_app1.pdf]

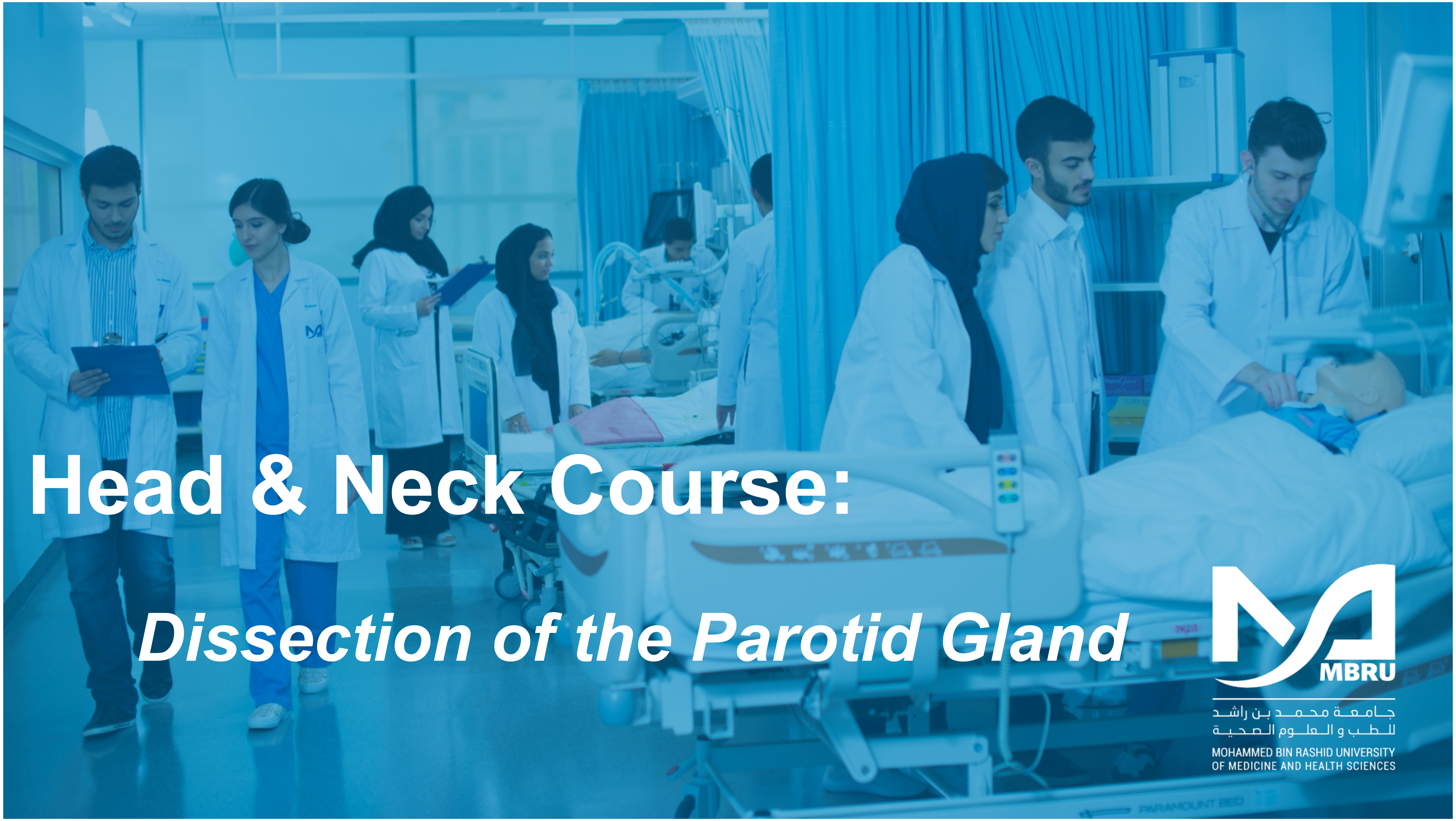

Head & Neck Course:

Dissection of the Parotid Gland

MBRU
جامعة محمد بن راشد
للطب و العلوم الصحية
MOHAMMED BIN RASHID UNIVERSITY
OF MEDICINE AND HEALTH SCIENCES

Learning Objectives:

At the end of this session, the student should be able to:

- Identify key surgical landmarks.
- Perform a safe resection of the parotid gland (i.e. total parotidectomy) during routine dissection by employing the detailed steps provided.

The dissection procedure of the parotid gland is described in 11 sequential steps:

Step 1:

Step 2:

Step 3:

Step 4:

Step 5:

Step 6:

Step 7:

Step 8:

Step 9:

Step 10:

Step 11:

POSITION:

Step 1:

- Place the cadaver in a supine position with the neck extended and turned to the opposite side.
- Ensure access is provided to the lobule of the ear superiorly, clavicle inferiorly, midline of the neck anteriorly and the trapezius muscle posteriorly.

Step 2:

Step 3:

Step 4:

Step 5: → Step 6: → Step 7: → Step 8: → Step 9: → Step 10: → Step 11:

PALPATION & IDENTIFICATION OF KEY ANATOMICAL LANDMARKS:

Step 1:

Step 2:

Step 3:

Step 4:

Step 5: → Step 6: → Step 7: → Step 8: → Step 9: → Step 10: → Step 11:

- **Palpate and identify the mastoid process, lobule of ear, preauricular region, angle of mandible and crease of neck.**

Step 1:

Step 2:

Step 3:

Step 4:

Step 5: → Step 6: → Step 7: → Step 8: → Step 9: → Step 10: → Step 11:

INCISION:

- **Make a modified Blair/S incision which will extend from the preauricular region, around the lobule of the ear towards the tip of the mastoid process, and then curve inferiorly beyond the angle of the mandible to finally join the neck crease.**

SKIN FLAP ELEVATION:

Step 1: → Step 2: → Step 3: → **Step 4:** → Step 5: → Step 6: → Step 7: → Step 8: → Step 9: → Step 10: → Step 11:

- Elevate the facial portion of the cervicofacial flap between the superficial musculoaponeurotic system and the parotid fascia to the boundary of the anterior border of the parotid gland.
- Observe the parotid fascia overlying the parotid gland.
- Note the shape of the parotid gland and identify the base and apex of it.

IDENTIFICATON OF GREAT AURICULAR NERVE:

- Identify the great auricular nerve and the external jugular vein at the anterior border of sternocleidomastoid muscle.
- Identify the anterior and posterior branches of the great auricular nerve.
- Dissect the posterior branch toward the ear lobe and preserve it.

Step 1:
Step 2:
Step 3:
Step 4:
Step 5:
Step 6:
Step 7:
Step 8:
Step 9:
Step 10:
Step 11:

Step 1:

Step 2:

Step 3:

Step 4:

Step 5: → Step 6: → Step 7: → Step 8: → Step 9: → Step 10: → Step 11:

SEPARATION OF PAROTID GLAND FROM STERNOCLEIDOMASTOID MUSCLE:

- **Outline the anterior border of the sternocleidomastoid muscle and bluntly dissect it from the parotid gland.**

IDENTIFICATON OF POSTERIOR BELLY OF DIGASTRIC MUSCLE:

- **At the apex, carefully reflect the parotid gland superiorly to expose the posterior belly of the digastric muscle.**

Step 1:
Step 2:
Step 3:
Step 4:
Step 5:
Step 6:
Step 7:
Step 8:
Step 9:
Step 10:
Step 11:

Step 1:

Step 2:

Step 3:

Step 4:

Step 5: → Step 6: → Step 7: → Step 8: → Step 9: → Step 10: → Step 11:

IDENTIFICATION OF LANDMARKS FOR FACIAL NERVE:

- **In order to locate the facial nerve, identify the tragal pointer, tympanomastoid suture, posterior belly of digastric and styloid process.**

Landmark	*Location of facial nerve in relation to landmark*
Tragal pointer	Approximately 1cm antero-infero-medial
Tympanomastoid suture	6-8mm deep to suture
Posterior belly of digastric muscle	Superior and parallel
Styloid process	Laterally-located

Step 1:

Step 2:

Step 3:

Step 4:

Step 5: → Step 6: → Step 7: → Step 8: → Step 9: → Step 10: → Step 11:

IDENTIFICATION OF PES ANSERINUS AND DIVISIONS OF FACIAL NERVE:

- **Identify the pes anserinus of the facial nerve.**
- **Follow the temporofacial and cervicofacial anteriorly.**

DISSECTION OF FACIAL NERVE FROM SUPERFICIAL LOBE OF PAROTID GLAND:

- Through blunt dissection, separate the superficial lobe (i.e. parotid fascia with parotid tissue) from the underlying facial nerve with the nerve always in sight. To ensure that the superficial lobe is completely stripped off the individual branches, sequentially dissect along each branch.

- Ensure that the division of the tissue does not extend beyond the exposed facial nerve.

- Distinguish between nerves and blood vessels as the superficial lobe is mobilized superiorly and eventually resected.

Step 1: → Step 2: → Step 3: → Step 4: → Step 5: → Step 6: → Step 7: → Step 8: → Step 9: → **Step 10:** → Step 11:

Step 1:

Step 2:

Step 3:

Step 4:

DISSECTION OF FACIAL NERVE FROM DEEP LOBE OF PAROTID GLAND:

- **Identify the superficial temporal vessels.**
- **In a circumferential manner, dissect and elevate the facial nerve and its branches from the underlying deep lobe.**
- **Completely resect the deep lobe of the parotid gland by detaching it from the posterior border of the mandible and the styloid process.**

Step 5: → Step 6: → Step 7: → Step 8: → Step 9: → Step 10: → Step 11:
